# Supplementary material for: Enhanced Lithium Storage Performance of α-MoO3/CNTs Composite Cathode
Source: Nanomaterials (Basel). 2023 Aug 7;13(15):2272. doi: 10.3390/nano13152272 (PMC10421027; doi:10.3390/nano13152272)
Supplement: Supplementary file 1 [file nanomaterials-13-02272-s001.zip › nanomaterials-2469437-supplementary.pdf]

Supporting Information:

Enhanced Lithium Storage Performance of α-MoO_3/CNTs Composite Cathode

Experimental

1.1. Materials synthesis

The α-MoO_{3-x}/CNTs composite was prepared via a simple one-step hydrothermal method. Firstly, the functionalized multi-walled CNTs were treated by adding 50 mL concentrated nitric acid and 1 g CNTs into a 100 mL Teflon-lined stainless-steel autoclave, and heated at 140 °C for 6 h. The CNTs from Shenzhen suiheng graphene technology, with a more than 99% purity. The multi-walled CNTs have an inner diameter of 3-5 nm, an outer diameter of 8-15 nm, and a length of 8-14 microns. The preparation method is CVD, and the particle size is less than 25 um. After the reaction, the functionalized CNTs were centrifuged and washed several times until the CNTs were neutral. Secondly, the treated functionalized CNTs were dispersed in 100 mL of deionized water and sonicated for 1 hour until the dispersion was uniform. Then 2.5 mol (3.089 g) ammonium molybdate tetrahydrate was added and sonicated for 1 hour, and 15 mL concentrated nitric acid was added and stir for 30 min. The mixture was poured into a 100 mL Teflon-lined stainless-steel autoclave, and the injected content was about 60 % of the lining. After sealing, the hydrothermal reaction was carried out at 180 °C for 24 h. After the reaction, it was naturally cooled to room temperature, washed with deionized water and anhydrous ethanol several times, and dried at 60 °C for 12 h to obtain α-MoO_{3-x}/CNTs composite powder. Finally, the α-MoO_3/CNTs free-standing film was obtained by vacuum filtration from the α-MoO_3/CNTs composite powder. Specifically, the α-MoO_3/CNTs composite powder was dispersed in 50 mL of deionized water. Then, the α-MoO_3/CNTs composite free-standing film was obtained by vacuum filtration of the powder dispersion. The pore size of the vacuum filtration membrane was 0.22 μm, and the mass load of the α-MoO_3/CNTs composite free-standing film of about 0.65 mg/cm^2. The preparation method of pure α-MoO_3 is the same as that of α-MoO_3/CNTs composites except that CNTs are not added.

1.2. Materials characterization

The morphology of the sample was observed using the scanning electron microscope (SEM, Hitachi, SU70) and transmission electron microscope (TEM, FEI, Tecnai TF20). The powder X-ray diffraction (XRD) experiments were conducted with Cu Kα (λ =1.5418 Å) using Japan Rigaku D/max-B X-ray diffraction. Raman spectra (LabRAMHR800, HORIBA Jobin Yvon, France) operating at the excitation wavelength of 532 nm laser. The α-MoO_3/CNTs composite was investigated by Thermogravimetric Analyzer (TG, Discovery TGA) at a temperature range of 50-700℃ under air with a ramp rate of 10 ℃/min. The valence state of the α-MoO_3/CNTs composite surface was investigated using X-ray photoelectron spectroscopy (XPS, Thermo Scientific ESCALAB Xi^+).

1.3. Electrochemical test

The α-MoO_3/CNTs composite and pure α-MoO_3 were used as a cathode for the lithium-ion battery for electrochemical tests. The α-MoO_3/CNTs composite and pure α-MoO_3 prepared by vacuum filtration were directly used as cathode electrodes without any conductive additives, binder, or current collector, and assembled into a coin cell (CR2032) for electrochemical performance testing.

The counter electrode is lithium metal. The electrolyte is 1.0 M LiPF6 dissolved in ethylene carbonate/dimethyl carbonate (EC/DMC=1:1 in volume), and the separator is Celgard2400. The galvanostatic charge-discharge measurements were conducted at room temperature in a battery-testing system (BTS-610, Netware China). Cyclic voltammetry (CV) and electrochemical impedance spectroscopy (EIS) were performed on an electrochemical workstation (CHI660E, CH Instruments, Inc.). Electrochemical impedance is tested in the frequency range of 100 kHz to 0.01 Hz. GITT test was carried out at a galvanostatic charge/discharge pulse of 50mA/g for 20 min.

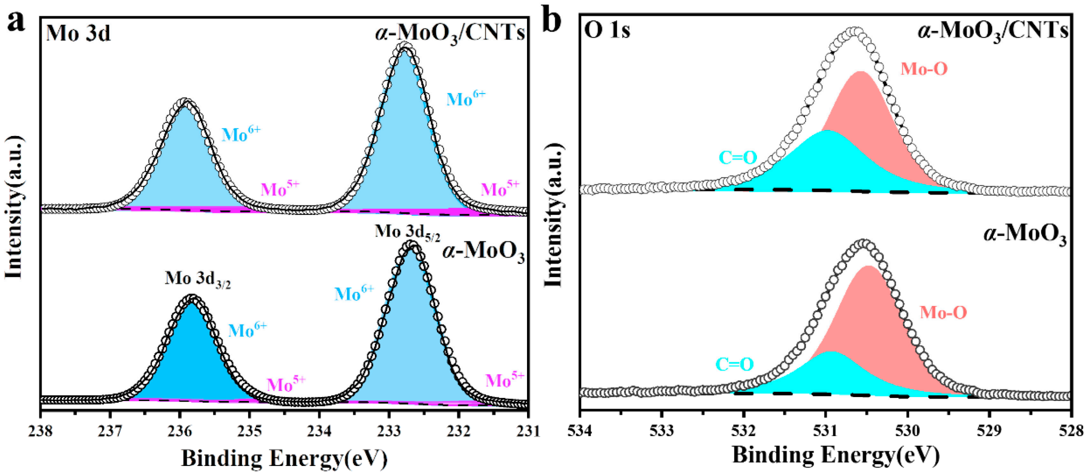

Figure S1. XPS spectra of the α-MoO_3/CNTs composite and pure α-MoO_3: (a) Mo 3d, (b) O 1s region.

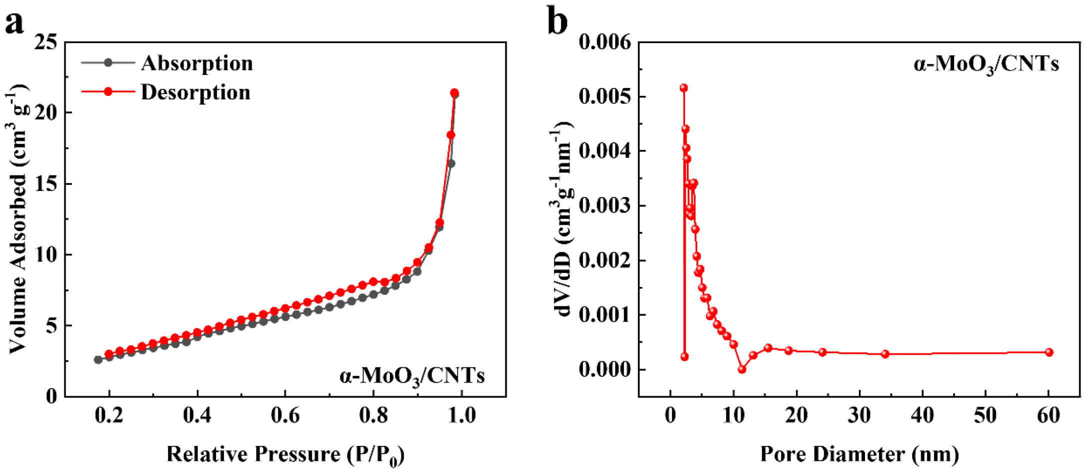

Figure S2. (a) N2 adsorption/desorption curve and (b) pore size distribution of the α-MoO_3/CNTs.
